# Gluteal muscles primary hydatid cyst after cortical bone destruction in the sacrum

**DOI:** 10.1016/j.amsu.2020.09.019

**Published:** 2020-09-12

**Authors:** Ammar Niazi, Abdelaziz Alibraheem, Ahmad Al-Mouakeh, Mohammad Karam Abouzied, Samer Rajab Basha, Shaban Suliman, Yasser Hendawi, Kusay Ayoub

**Affiliations:** a Department of surgery, Faculty of medicine, University of Aleppo, Syria; b Faculty of medicine, University of Aleppo, Aleppo, Syria

**Keywords:** Hydatid disease, Sacrum, Echinococcosis, Paresthesia, Gait disorders, Main text

## Abstract

**Introduction:**

Hydatid disease is caused by infection of Echinococcus Granulosus. Usually Hydatid Cysts occur in the liver and lungs. Presenting hydatid cysts in bone without hepatic affectation is rare and occurs in 0.5–2% of cases. Hence, this rare case makes the diagnosis difficult for the clinicians and, as a result, misdiagnosis of sacral Echinococcosis is common.

**Presentation of case:**

The authors report on a 47-year male with primary sacral hydatidosis and 34 years of recurrence. He was admitted with compressive neurological symptoms like tingling pain, numbness, sciatica and foot drop. He has undergone 8 operations and has been treated with Albendazole. He has developed a Sacro-cutaneous fistula.

**Discussion:**

When assessing sciatica, low back pain or lower limb weakness the pelvic cavity should be examined for hidden disease that might explain the neurological symptoms.

**Conclusion:**

A missed diagnosis of osseous Hydatidosis could be devastating. Accordingly, the sacral Hydatid cyst must be included as a differential diagnosis for compressive neurological symptoms. In clinical practice, surgery remains the gold standard for treating osseous Hydatidosis.

## Highlights

• The symptoms of osseous echinococcosis are non-specific and widely variable.
• Sacral hydatid cyst it should be included in the differential diagnosis of any bone mass caused compressive neurological symptoms.
• Treatment of hydatid cyst is primarily surgical, demanding total removal without rupture.
• Posterior approach is preferred over anterior due to feasibility of the intervention and decrease of the recurrence rate.

## 1 Introduction

Our paper has been reported with the SCARE 2018 criteria [9].

The larval stage of the cystoid Echinococcus Granulosus causes Hydatid cysts, and it is a zoonotic infestation for which humans are intermediate host. It mainly found in the liver (~70%) and the lungs (~20%), but it can be found in all the body, including the bone (~0.5–4%) of all cases of Echinococcosis described in the literature [1], cases which involves the spine occurs in about 50% of the cases of bone hydatid disease [2]. The cysts are usually placed in the thoracic (52%) and lumbar (37%) levels [3]. More rarely, hydatid cysts are found at the cervical and sacral regions (11%) [3].

The infestation is prevalent in most parts of the world, especially in sheep farming and cattle farming areas of the Mediterranean Sea, Asia, North and East Africa, South America, Australia, and the Middle East.

Spinal echinococcosis’ symptoms are mostly related to pressure exerted by the cyst itself on nerve roots, leading to neurological manifestations of peripheral sensitivity loss, radicular pain, paraparesis, paraplegia, sphincter disturbance or bladder dysfunction [4].

## 2 Case report

A 47-year male was referred to our surgical clinic in 2006 with back pain, numbness in left leg, perineum. Neurological examination revealed perianal hypoesthesia and gradual paraparesis in the left muscle group with no bladder disorders.

Neurological examination showed a weakness of the lower extremities, bilateral hypotonia and moderate flexion contracture of the left ankle. The Babinski response was positive.

Physical examination showed that his reflex test was normal. Due to his complaint, the patient does not have a normal gait, he depends more on his right leg and avoids prolonged pressure on the left one. His symptoms were initially misdiagnosed in 1985 as Ankylosing Spondylitis and treated with for 5 years accordingly.

**Fig. 1:**
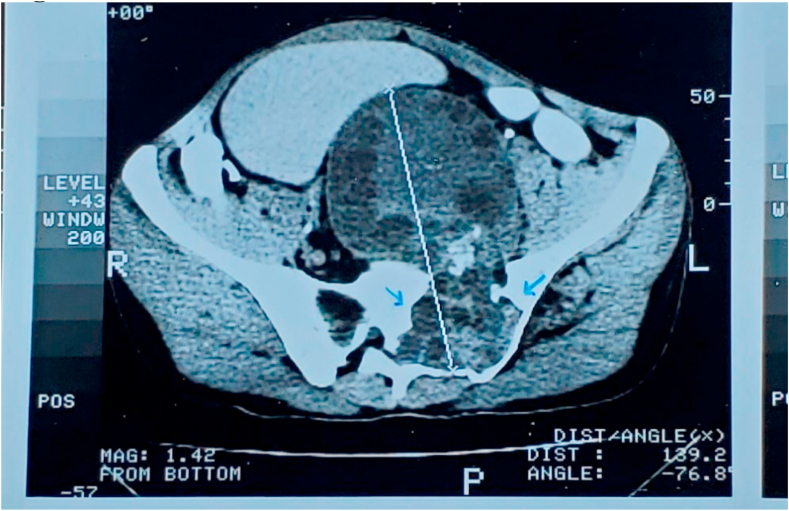
An expansile lytic lesion affecting the left iliac wing and the sacrum with cortical bone destruction.

His diagnosis of Hydatid Cyst was first made in 1990 by Abdominopelvic Ultrasonography that showed an expansive lytic lesion affecting the left iliac wing and the sacrum with cortical bone destruction and was later confirmed by pelvic CT scan (computed tomography) that revealed multiple expansive osteolytic lesions containing trabeculae with cortical thinning located in the left sacral ala and spreading to the left iliac bone. (And showed cystic enlargement in the left pelvis over the lumbosacral plexus roots (Fig. 1).

**Fig. 2:**
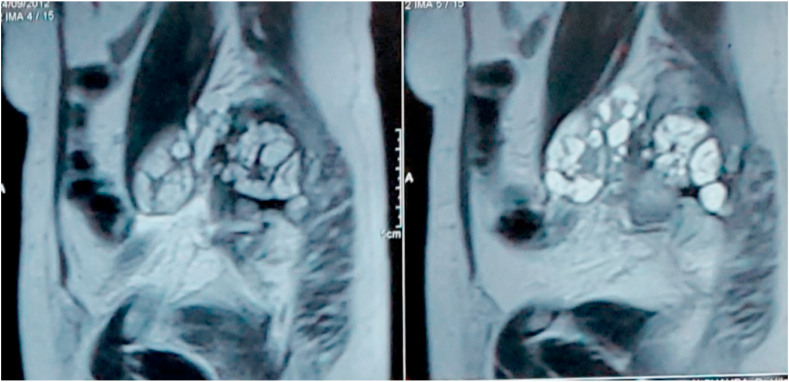
Sacral destruction and replacement by a multiloculated cystic mass.

Recent CT Imaging showed cysts expand to nearby regions in the gluteal muscles as well. MRI (magnetic resonance imaging) there was an expansible lesion with high signal intensity in T2-weighted images and low signal intensity in T1-weighted images, sacral destruction and replacement by a multiloculate cystic mass (Fig. 2). Clinical and radiologic examination did not show any location of disease other than the spine. Weinberg and other Serological tests were positive as well.

The treatment regimen included both surgical decompression of the lesion site and medical prescription of Albendazole.

The patient had his first surgical procedure in 1995 with year-long physiotherapy, the patient remained asymptomatic for four years. Then, two operations were done in 1999 and 2003 accompanied by the deterioration of mobility in the left lower limb as signs of drop foot have appeared. Five later operations dating in 2006, 2009, 2012, 2015 and 2018, were performed with an orthopedic surgeon due to change in intervention's initial site “posterior intervention”.

**Fig. 3:**
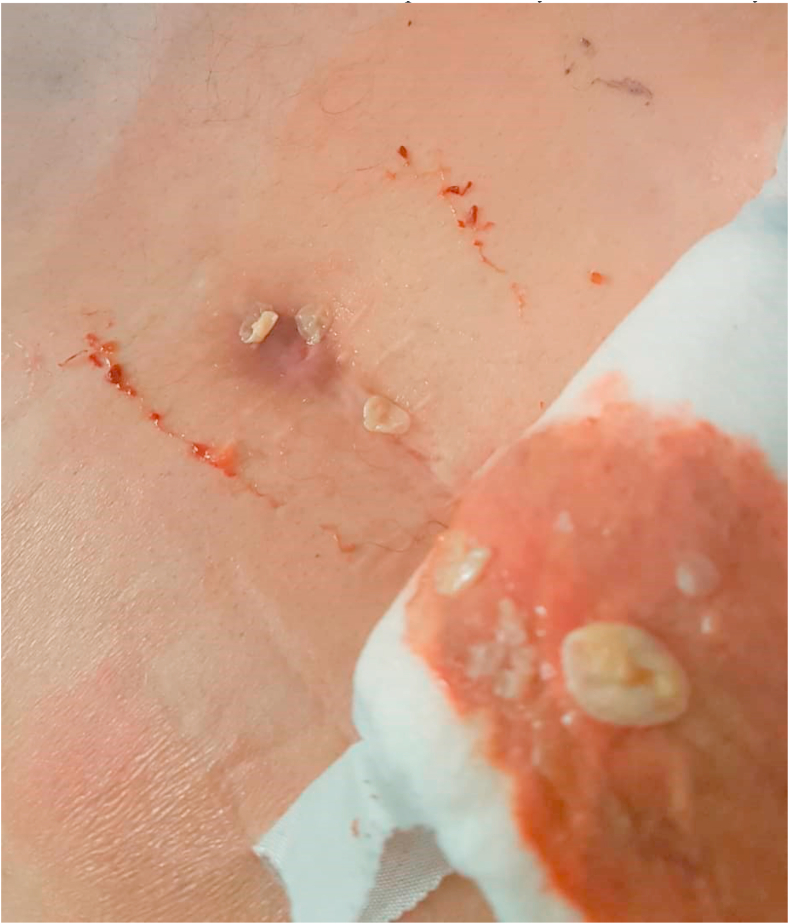
Sacro-cutaneous fistula, through which daughter cysts would spring up.

Following the last operation in 2018, the Patient has developed a Sacro-cutaneous fistula, through which daughter cysts would spring up (Fig. 3). After each operation, the patient has been prescribed on Albendazole on a 3-course method, each course lasting for 28 days, during which the patient takes 100 mg of Albendazole 2 times a day by oral administration. Each course was followed by 3 weeks rest period.

Paraparesis and perianal hypoesthesia were improved almost completely in later controls extending over 3 years. The cutaneous fistula allowed the cysts to exit continuously and prevented them from gathering.

## 3 Discussion

Hydatid disease is a well-known condition that affects liver and lungs. And although it is rarely affects the bone, it should be included in the differential diagnosis of any bone mass caused compressive neurological symptoms [5].

When the bone is affected by hydatid cysts, it doesn't form encapsulated cysts like in other organs [6]. For this reason, diagnosis may be delayed. Surgical treatment is proved to be the gold standard and radical surgery is well-advised.

Although hydatid disease of bone is rare, it should be included in the differential diagnosis of any bone mass discovered in the human body.

In a systematic review of 721 cases of Osseous echinococcosis, 95% of cases required one intervention, although in other cases up to four interventions were performed. Notably, complete excision of the lesion was only accessible in 16% of cases. The two main primary procedures in Spinal Echinococcosis would be decompression of the affected spinal root and stabilization of column following resection [7].

In order to decrease recurrence, intraoperative irrigation with scolicidal solutions is advised. This can be performed using hypertonic saline, cetrimide, silver nitrate, sodium hypochlorite or glycerin [8].

Formaldehyde was avoided in the last 5 surgical procedures due to its irritation features on root nerves being exposed using the posterior approach. This method was preferred over anterior approach due to feasibility of the intervention and decrease of the recurrence rate.

In our case, Diagnosis was delayed because the pelvic cavity was not studied when radiculopathy symptoms started and the patient did not interact with any of the animals in his history.

Recurrence is referred to the spongy characteristic of the sacral bone as those small spaces would allow seeding of the daughter cysts and are not accessible through surgical intervention.

We noticed eruption of hydatid cysts through the Sacro-cutaneous fistula after exertion, such as holding heavy objects. This can be interpreted by the existence of hydatid cysts in the iliopsoas muscle so that any contraction of the muscle would cause the cysts to come out. We noticed closure of the fistula and relief of the pain when the patient does not exert much effort.

## 4 Conclusion

Treatment of hydatid cyst is primarily surgical, demanding total removal without rupture.

In areas where hydatid disease is an endemic, sacral hydatid cyst it should be included in the differential diagnosis of any bone mass caused compressive neurological symptoms for early and correct treatment in order to decrease recurrence. In this case report we identified a case of recurrent osseous hydatidosis that was initially misdiagnosed which in turn led to mistreatment.

## Provenance and peer review

Not commissioned, externally peer reviewed.

## Declaration of competing interestCOI

Authors declare that there is no conflict of interest.

## Sources of funding

This research did not receive any specific grant from funding agencies in the public, commercial, or not-for-profit sectors.

## Ethical approval

We have the patient’s approval; no more approvals are required. The work has not been published previously.

## Consent

Written informed consent was obtained from the patient for publication of this case report and accompanying images. A copy of the written consent is available for review by the Editor-in-Chief of this journal on request.

## Author contribution

Ammar Niazi and Kusay Ayoub diagnosed and treated the patient, Abdelaziz Alibraheem and Yasser Hendawi searched the literature, Mohammad Karam Abouzied, Samer Rajab Basha and Shaban Suliman wrote the manuscript and Abdelaziz Alibraheem and Ahmad Al-Mouakeh critically revised the article. All authors approved the final version of the manuscript.

## Guarantor

Abdelaziz Alibraheem

Email: abdelazizalibraheem@gmail.com
