# Supplementary material for: Gluteal muscles primary hydatid cyst after cortical bone destruction in the sacrum
Source: Ann Med Surg (Lond). 2020 Sep 12;59:89–92. doi: 10.1016/j.amsu.2020.09.019 (PMC7501496; doi:10.1016/j.amsu.2020.09.019)
Supplement: Multimedia component 1 [file mmc1.docx]

| **SCARE 2018 Checklist** | | | |
| --- | --- | --- | --- |
| **Topic** | **Item** | **Checklist item description** | **Page Number** |
| **Title** | **1** | Gluteal Muscles Primary Hydatid Cyst After Cortical Bone Destruction in The Sacrum | 1 |
| **Key Words** | **2** | **hydatid disease; sacrum; echinococcosis; Paresthesia;**  **Gait Disorders; case report** | 1 |
| **Abstract** | **3a** | Hydatid cyst in the sacrum is rare. this rare case makes the diagnosis difficult for the clinicians and, as a result, misdiagnosis of sacral Echinococcosis is common. | 1 |
|  | **3b** | Patient was admitted with compressive neurological symptoms like tingling pain, numbness, sciatica and foot drop. He has undergone 8 operations and has been treated with Albendazole. He has developed a Sacro-cutaneous fistula. |  |
|  | **3c** | The first diagnosis was Ankylosing Spondylitis and treated with for 5 years accordingly.  Then the diagnosis was edited to sacral hydatid cyst and was treated by 8 operation and with albendazole. |  |
|  | **3d** | A missed diagnosis of osseous Hydatidosis could be devastating. Accordingly, the sacral Hydatid cyst must be included as a differential diagnosis for compressive neurological symptoms. In clinical practice, surgery remains the gold standard for treating osseous Hydatidosis. |  |
| **Introduction** | **4** | The larval stage of the cystoid Echinococcus Granulosus causes Hydatid cysts, and it is a zoonotic infestation for which humans are intermediate host. It mainly found in the liver (~70%) and the lungs (~20%), but it can be found in all the body, including the bone (~0.5-4%) of all cases of Echinococcosis described in the literature[1], cases which involves the spine occurs in about 50% of the cases of bone hydatid disease[2]. The cysts are usually placed in the thoracic (52%) and lumbar (37%) levels[3]. More rarely, hydatid cysts are found at the cervical and sacral regions (11%)[3].  Spinal echinococcosis’ symptoms are mostly related to pressure exerted by the cyst itself on nerve roots, leading to neurological manifestations of peripheral sensitivity loss, radicular pain, paraparesis, paraplegia, sphincter disturbance or bladder dysfunction[4]. | 2 |
| **Patient Information** | **5a** | Male patient with 47-year-old | **2** |
|  | **5b** | The patient was referred to our institute in 2006 with back pain, numbness in left leg, perineum. Neurological examination revealed perianal hypoesthesia and gradual paraparesis in the left muscle group with no bladder disorders.  The patient does not have a normal gait, he depends more on his right leg and avoids prolonged pressure on the left one. |  |
|  | **5c** | The patient was come to our institute in 2006 after 2 operation and recurrence. |  |
|  | **5d** | The patient dose not smoking and his pharmacological and family history was clear.  The patient mentioned of having a cat since he was young. |  |
| **Clinical Findings** | **6** | Neurological examination revealed perianal hypoesthesia and gradual paraparesis in the left muscle group with no bladder disorders.  His reflex test was normal | 2 |
| **Timeline** | **7** | His symptoms were initially misdiagnosed in 1985 as Ankylosing Spondylitis and treated with for 5 years accordingly.  His diagnosis of Hydatid Cyst was first made in 1990 by Abdominopelvic Ultrasonography.  The patient had his first surgical procedure in 1995 with year-long physiotherapy, the patient remained asymptomatic for four years. Then, two operations were done in 1999 and 2003 accompanied by the deterioration of mobility in the left lower limb as signs of drop foot have appeared. Five later operations dating in 2006, 2009, 2012, 2015 and 2018, were performed with an orthopedic surgeon due to change in intervention’s initial site “posterior intervention”. | 2 |
| **Diagnostic Assessment** | **8a** | Diagnostic methods: His diagnosis of Hydatid Cyst was first made in 1990 by Abdominopelvic Ultrasonography.  Then with CT scan (computed tomography) and with MRI (magnetic resonance imaging). | 2 |
|  | **8b** | Diagnostic challenges: this case was diagnosis wrong and the reason was this case is rare and the hydatid cyst was in the sacral bone and this pone have spongy characteristic. |  |
|  | **8c** | Diagnostic reasoning with Abdominopelvic Ultrasonography and CT scan (computed tomography) and with MRI (magnetic resonance imaging) the diagnosis was clear as hydatid cyst in sacral bone. |  |
|  | **8d** | An expansile lytic lesion affecting the left iliac wing and the sacrum with cortical bone destruction.  Sacral destruction and replacement by a multiloculated cystic mass. |  |
| **Therapeutic Intervention** | **9a** | Pre-intervention considerations: His symptoms were initially misdiagnosed in 1985 as Ankylosing Spondylitis and treated with for 5 years accordingly.  After hydatid cyst was diagnosed the first precure was surgical. | 2 |
|  | **9b** | Interventions: 8 operation from 1990 until 2018 and after that the patient had a cutaneous fistula. |  |
|  | **9c** | Intervention details: The treatment regimen included both surgical decompression of the lesion site and medical prescription of Albendazole.  The patient had his first surgical procedure in 1995 with year-long physiotherapy, the patient remained asymptomatic for four years. Then, two operations were done in 1999 and 2003 accompanied by the deterioration of mobility in the left lower limb as signs of drop foot have appeared. Five later operations dating in 2006, 2009, 2012, 2015 and 2018, were performed with an orthopedic surgeon due to change in intervention’s initial site “posterior intervention”. |  |
|  | **9d** | Who performed the procedure: Ammar Niazi, PHD, Department of surgery, Faculty of medicine, University of Aleppo |  |
|  | **9e** | Changes: The patient had his first surgical procedure in 1995 with year-long physiotherapy, the patient remained asymptomatic for four years. Then, two operations were done in 1999 and 2003 accompanied by the deterioration of mobility in the left lower limb as signs of drop foot have appeared. Five later operations dating in 2006, 2009, 2012, 2015 and 2018, were performed with an orthopedic surgeon due to change in intervention’s initial site “posterior intervention”. |  |
|  | **9f** | Following the last operation in 2018, the Patient has developed a Sacro-cutaneous fistula, through which daughter cysts would spring up (figure3). After each operation, the patient has been prescribed on Albendazole on a 3-course method, each course lasting for 28 days, during which the patient takes 100 mg of Albendazole 2 times a day by oral administration. Each course was followed by 3 weeks rest period. |  |
| **Follow-up and**  **Outcomes** | **10a** | Follow-up – describe 1) from 2006 until now. 2) in Department of surgery, Faculty of medicine, University of Aleppo. 3) After each operation, the patient has been prescribed on Albendazole on a 3-course method, each course lasting for 28 days, during which the patient takes 100 mg of Albendazole 2 times a day by oral administration. Each course was followed by 3 weeks rest period. | 2 |
|  | **10b** | Outcomes: the Patient has developed a Sacro-cutaneous fistula, through which daughter cysts would spring up |  |
|  | **10c** | Intervention adherence/compliance: After each operation, the patient has been prescribed on Albendazole on a 3-course method, each course lasting for 28 days, during which the patient takes 100 mg of Albendazole 2 times a day by oral administration. Each course was followed by 3 weeks rest period. |  |
|  | **10d** | Complications and adverse events: firstly, the case was misdiagnosed because of its rare.  Secondly, the recurrence is referred to the spongy characteristic of the sacral bone as those small spaces would allow seeding of the daughter cysts and are not accessible through surgical intervention. |  |
| **Discussion** | **11a** | Strengths: hydatid cyst in sacrum is rare and it should be included in the differential diagnosis of any bone mass caused compressive neurological symptoms for early and correct treatment in order to decrease recurrence.  the recurrence is referred to the spongy characteristic of the sacral bone | 3 |
|  | **11b** | Weaknesses and limitations in your approach to this case. We think hydatid cyst in sacrum is rare and it should be included in the differential diagnosis of any bone mass caused compressive neurological symptoms especially in areas where hydatid disease is an endemic  And although the accuracy of the surgical procedure the recurrence is the role because of the spongy characteristic of the sacral bone |  |
|  | **11c** | In a systematic review of 721 cases of Osseous echinococcosis, 95% of cases required one intervention, although in other cases up to four interventions were performed. Notably, complete excision of the lesion was only accessible in 16% of cases. The two main primary procedures in Spinal Echinococcosis would be decompression of the affected spinal root and stabilization of column following resection[7]. |  |
|  | **11d** | After looking in systematic review and other studies we notice that sacral hydatid cyst maybe treatment without recurrence if demanding total removal without rupture |  |
|  | **11e** | Treatment of hydatid cyst is primarily surgical, demanding total removal without rupture. |  |
| **Patient Perspective** | **12** | When appropriate the patient should share their perspective on the treatments they received. | 8 |
| **Informed Consent** | **13** | informed consent was obtained. | 8 |
| **Additional Information** | **14** | Ethics and consent to participate: We have the patient’s approval; no more approvals are required. The work has not been published previously.  Consent to publish: Written informed consent was obtained from the patient for publication of this Case Report and any accompanying images. A copy of the written consent is available for review by the Editor of this journal.  Competing interests: Authors declare that there is no conflict of interest.  Availability of data and materials: All data and materials are available. | 8 |
